# Editorial: Predictive Imagable Biomarkers for Neurodegenerative and Neurodevelopmental Diseases

**DOI:** 10.3389/fneur.2019.00583

**Published:** 2019-06-13

**Authors:** Pravat K. Mandal, Lars Ersland

**Affiliations:** ^1^Neuroimaging and Neurospectroscopy Laboratory (NINS), National Brain Research Centre, Gurgaon, India; ^2^Florey Institute of Neuroscience and Mental Health, Melbourne, VIC, Australia; ^3^Department of Clinical Engineering, Haukeland University Hospital, Bergen, Norway; ^4^Department of Biological and Medical Psychology, University of Bergen, Bergen, Norway; ^5^NORMENT Center of Excellence, Haukeland University Hospital, Bergen, Norway

**Keywords:** imagable biomarkers, neurodevelopmental, neurodegenerative, glutathione conformations, imaging techniques

In the last four decades, tremendous economic and technological development has helped to improve the quality of life and average life span has increased substantially. As a consequence, the number of people with much higher age is increasing and reports of aging associated disorders are multiplying due to various neurodegenerative disorders such as Alzheimer's, Parkinson's frontotemporal, dementia with Lewy body disease etc. The causal process of these neurodegenerative disorders is not known yet; however, oxidative stress is recognized to play an important role (1–3). At the same time, the number of cases with neurodevelopmental disorders [Autism Spectrum Disorders (ASD) (4), Epilepsy (5) and Attention Deficit Hyperactivity Disorder (6)] is increasing rapidly in the early part of the life due to multifactorial reasons. Two major health related issues in two distinct age groups need urgent attention to identify the causal process and subsequently a therapeutic development for cure.

The advancements in different imaging techniques [e.g., Magnetic Resonance Imaging (MRI), MR Spectroscopy (MRS), functional MRI (fMRI), functional MRS, Magnetic Encephalography, Diffusion Tensor Imaging etc.] provide various critical features for reliably predicting the individuals who will progress from asymptomatic pre-clinical phase to clinical phases.

In this context, it is critical to investigate the factors which may impact the brain microenvironment these could trigger the early causal processes. In neurodevelopmental and neurodegenerative disorders, the roles of various neurochemicals receptors or antioxidants and their abnormal modulations are getting huge attention for more in-depth research. Recently it was discovered that brain microenvironment has the role to modulate the two distinct conformations of glutathione (GSH), a major antioxidant involved in neutralizing harmful radicals (Figure 1). GSH exists in two conformational states (extended and closed form) in the brain (7, 10, 11). It is therefore paramount to identify novel imagable diagnostic biomarkers involving antioxidants, neurotransmitters and physiological parameters that can aid in discovering the causal processes of these brain disorders and can be translated into clinical practices for simplified diagnostic tests and advocating appropriate lifestyle changes to delay the onset of symptoms.

**Figure 1:**
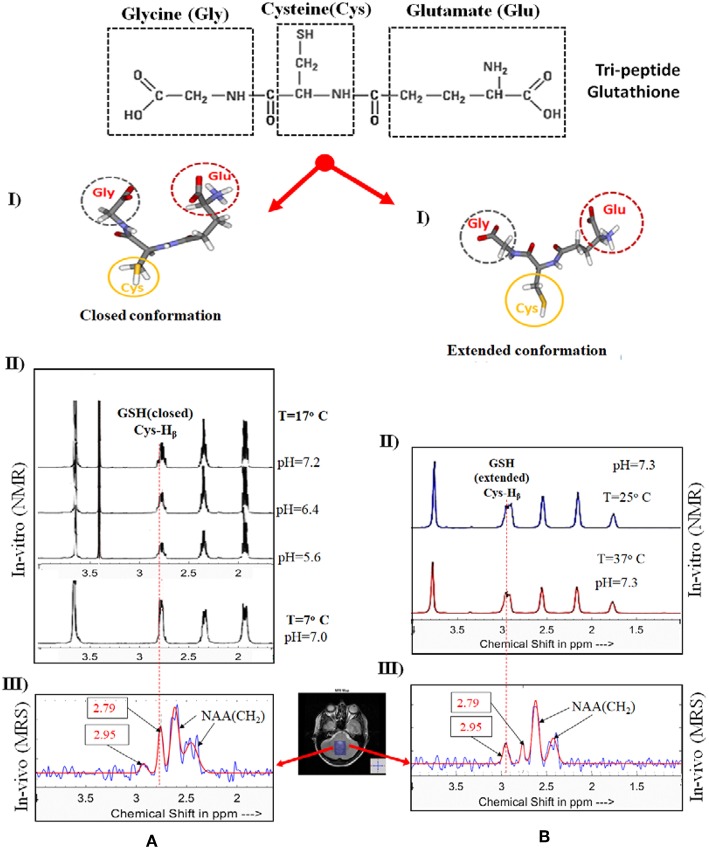
Impact of brain microenvironment on the conformational changes of GSH as evidenced by *in vitro* and *in vivo* MR spectroscopy. Tripeptide GSH is available in two forms. In the panel **(A)**, (I and II), represents the structure of closed form of GSH and the NMR spectra. The second panel **(B)**, (I and II), presents the extended conformation of GSH form and the NMR spectra. Necessary permissions [processed using KALPANA package (1)] were taken to reproduce GSH structure and the NMR spectra from the publishers [IOS PRESS (7), FEBS (8) and Springer (9)]. **(A,B)** (III) display MEGA-PRESS spectra of the cerebellum of the same subject for closed form of GSH (with 180° excitation pulse at 4.40 ppm and TE/TR 130/2500 ms) and extended GSH form (with selective 180° pulse applied at 4.56 ppm and TE/TR 130/2500 ms) using 3T Philips Achieva at NINS Lab, NBRC, India). The NMR spectrum of GSH (closed and extended forms) is aligned with MR spectra of GSH from human brain.

This special issue has a total of ten articles from various laboratories. Mandal and co-workers have developed a Hadoop-based big data framework (BHARAT) integrating non-invasive MRI, MRS as well as neuropsychological test outcomes to identify early diagnostic biomarkers of AD. The framework for AD incorporates the three “V”s (volume, variety and velocity) with advanced data mining, machine learning, and statistical modeling algorithms Sharma et al.

Feng et al. have used corpus callosum (CC) radiomic features related to the diagnosis of AD. They have aimed to identify the CC radiomic features related to the diagnosis of AD and build classification model based on machine learning for the diagnosis of AD.

Teipel et al. have used MRI and cognitive data from 124 patients, derived from ANDI-1 cohort (follow up period 0.4–3.1 years). They conclude that basal forebrain volume, but not hippocampus volume, is a significant predictor of rates for global cognitive decline to predict subsequent cognitive decline during cholinergic treatment.

Yamasakhi et al. have studied the driving ability in the Alzheimer's Disease Spectrum (ADS) and have hypothesized that feasibility of event-related potentials can be a possible predictive biomarker of driving ability in ADS. Interestingly, even in the early stage of the disease, patients with ADS are characterized by the impairment of visuospatial function such as radial optic flow perception related to self-motion perception.

Oishi et al., using functional MRI, found a significant relationship between low Gray Matter (GM) volume in the right inferior parietal lobule (IPL) and severity of mental disorientation. They hypothesize that right IPL is responsible for mental disorientation in amnestic MCI (aMCI) based on voxel-based morphometry. A significant decreased GM volume has been found in the right IPL, which correlates with lower orientation scores on the COGNISTAT cognitive testing tool.

O'Gorman Tuura et al. have investigated the relationship between the axial symptoms of PD and GABA and glutamate levels have quantified using MRS (PD patients *N* = 20 and 17 healthy control). The study showed associations between GABA and Glx and axial symptoms scores are typically more prominent in akinetic-rigid patients than in tremor-dominant patients.

Emamzadeh et al. have presented various risk factors for PD and PD treatment options. Potential risk factors include environmental toxins, drugs, pesticides, brain microtrauma, focal cerebrovascular damage, and genomic defects. Conventional pharmacological treatment of PD is based on the replacement of dopamine using dopamine precursors (levodopa, L-DOPA, L-3,4 dihydroxyphenylalanine), dopamine agonists (amantadine, apomorphine) and MAO-B inhibitors (selegiline, rasagiline), which can be used alone or in combination with each other.

Amyotrophic lateral sclerosis (ALS) is a progressive neurodegenerative process affecting upper and lower motor neurons as well as non-motor systems. Wirth and corworkers report the precentral and postcentral cortical thinning detected by structural MRI combined with clinical (ALS-specific functional rating scale revised, ALSFRS-R) and neurophysiological (motor unit number index, MUNIX) biomarkers in both cross-sectional and longitudinal analyses. Their study concludes that a combinatory use of structural MRI, neurophysiological and clinical biomarkers allows for an appropriate and detailed assessment of clinical state and course of disease of ALS Wirth et al.

Dwyer et al. have reported that no significant changes in GABA, Glx, or NAA levels are observed as a result of anodal stimulation, or between active and sham stimulation, suggesting that a single session of anodal tDCS to the pSTG may be less effective than in other cortical areas.

Winklewski et al. report that DTI can reveal strategic information with respect to white matter tracts, disconnection mechanisms, and related symptoms. Axial and radial diffusivity are likely to provide quite consistent information in healthy subjects, and in pathological conditions with limited edema and inflammatory changes. DTI remains one of the most promising non-invasive diagnostic tools in medicine.

It is our sincere efforts to bring the latest research from leading laboratories to enrich the area, and we believe that multi-centric research collaboration could immensely help to identify various factors responsible for brain microenvironment changes. These critical features can be used in big data analytics, and it should subsequently help in setting a successful clinical trial (9, 12).

## Author Contributions

PKM conceptualized the idea and wrote the first draft. LE discussed and participated in the draft finalization. The final version was approved by both authors.

### Conflict of Interest Statement

The authors declare that the research was conducted in the absence of any commercial or financial relationships that could be construed as a potential conflict of interest.
